# Monitoring Maize Lodging Grades via Unmanned Aerial Vehicle Multispectral Image

**DOI:** 10.34133/2019/5704154

**Published:** 2019-12-31

**Authors:** Qian Sun, Lin Sun, Meiyan Shu, Xiaohe Gu, Guijun Yang, Longfei Zhou

**Affiliations:** ^1^National Engineering Research Center for Information Technology in Agriculture, Beijing, China; ^2^College of Geomatics, Shandong University of Science and Technology, Qingdao, China; ^3^College of Resources and Environmental Sciences, China Agricultural University, Beijing, China

## Abstract

Lodging is one of the main factors affecting the quality and yield of crops. Timely and accurate determination of crop lodging grade is of great significance for the quantitative and objective evaluation of yield losses. The purpose of this study was to analyze the monitoring ability of a multispectral image obtained by an unmanned aerial vehicle (UAV) for determination of the maize lodging grade. A multispectral Parrot Sequoia camera is specially designed for agricultural applications and provides new information that is useful in agricultural decision-making. Indeed, a near-infrared image which cannot be seen with the naked eye can be used to make a highly precise diagnosis of the vegetation condition. The images obtained constitute a highly effective tool for analyzing plant health. Maize samples with different lodging grades were obtained by visual interpretation, and the spectral reflectance, texture feature parameters, and vegetation indices of the training samples were extracted. Different feature transformations were performed, texture features and vegetation indices were combined, and various feature images were classified by maximum likelihood classification (MLC) to extract four lodging grades. Classification accuracy was evaluated using a confusion matrix based on the verification samples, and the features suitable for monitoring the maize lodging grade were screened. The results showed that compared with a multispectral image, the principal components, texture features, and combination of texture features and vegetation indices were improved by varying degrees. The overall accuracy of the combination of texture features and vegetation indices is 86.61%, and the Kappa coefficient is 0.8327, which is higher than that of other features. Therefore, the classification result based on the feature combinations of the UAV multispectral image is useful for monitoring of maize lodging grades.

## 1. Introduction

Maize (Zea mays L.) is an important food and feed crop, which is mainly distributed at latitudes 30°-50°, and is also the highest yielding crop in the world. The north, northeast, and southwest mountain areas of China are the main maize-producing regions. Maize is a thermophilic crop, which requires high temperature during the whole growth period. During the late growth stage of maize, strong winds and heavy rainfall, as well as the structural characteristics of maize plants such as tall and weaker stems, increase the likelihood of large-scale lodging [1–4]. Lodging hinders maize growth, leading to a decrease in yield and a reduction in grain quality [4, 5]. Timely and accurate acquisition of information with different lodging grades of maize will help agricultural management departments and insurance companies to calculate area and yield losses, to perform postdisaster production management and relief work.

Accurate acquisition of information on maize lodging relies on conventional means, such as artificial field investigations and sample measurements, which are time-consuming, labor-intensive, and inefficient. The rapid development of remote sensing technology provides new methods for obtaining information on crop lodging. A method based on remote sensing has advantages of low cost and high convenience and is widely used to extract phenotypic information about crops [6, 7]. A maize lodging model based on the difference of the ratio vegetation index (RVI) before and after lodging can be constructed using HJ-1B CCD multispectral images [8]. Hyperspectral remote sensing with principal component analysis (PCA) and artificial neural networks may be applied to discriminate lodged from normal crops on regional and large scales [9]. With improved remote sensing monitoring capability, the ultralow altitude UAV system has become a focus for crop lodging investigation because of advantages such as its low cost, high resolution, and ability to acquire images under clouds. Red-green-blue (RGB) images were acquired by a small UAV equipped with a digital camera to analyze the image features of nonlodging and lodging maize, and a method for extracting the maize lodging area based on color and texture features was established [10]. Liu et al. [11] analyzed the spectral characteristics and texture features of wheat lodging based on UAV data and adopted an object-oriented method to extract the lodging area of wheat. Based on the digital surface model (DSM) and texture information obtained from the UAV image, Yang et al. [12] reported that the optimal model was a decision tree classification combined with the single feature probability (SFP) value. A method was proposed by Chu et al. [13] to evaluate the lodging severity of maize fields using UAV digital cameras and information on plant height. The genetic factors affecting maize lodging and predicting the lodging rate were quantitatively identified based on UAV digital and multispectral images by analyzing the nomograms [14]. Wilke et al. [15] quantified the lodging rate and severity based on the UAV canopy height model combined with the target threshold method and evaluated the potential application of UAV images in quantitative research on the lodging rate and severity using structure from motion (SfM). The differential digital elevation model of unmanned aerial systems (UAS) was used for the quantitative evaluation and verification of wheat lodging. Alternative visual evaluation of UAS-based crop high-throughput phenotypes was found to be important role for complex lodging phenological characteristics [16].

To date, many studies have focused on extracting lodging information and area by using RGB image, but relatively few studies have further subdivided lodging grades. RGB image contains only three bands of data representing the intensities of red, green, and blue wavelengths of each pixel [17, 18]. It covers less crop information and can only obtain the image information of ground objects. A multispectral imager with a near-infrared band is an important sensor widely used in agricultural remote sensing from a satellite platform to a near ground platform, which can obtain both image information and spectral information of ground objects at the same time. There are few studies on the spectral characteristics of crops in the existing research on crop lodging monitoring based on UAV. The spectral characteristics, especially the red edge and infrared band, can reflect the growth status of crops from the essential aspects such as physical and chemical properties and canopy structure [19, 20]. There is a clear physical significance to monitor crop lodging on the basis of spectral characteristics. Numerous studies [21–23] have shown that the lodging crop has stronger reflectance from leaves and stalks in the NIR band which results in the large contrast between the lodging and nonlodging areas. Parrot Sequoia is tailor-made for agriculture and can be used in a variety of agricultural applications with application width and depth. UAV with a Sequoia multispectral camera is used to obtain farmland images and capture information from visible and invisible light in order to measure the health and vitality of crops. Based on a multispectral image with a spatial resolution of 0.05 m obtained by UAV on September 10, 2018, the spectral characteristics of the canopy of different lodging grades were analyzed at the maize filling stage. Principal component transformation was performed, the texture features were extracted, the vegetation indices were constructed, and the maximum likelihood classification (MLC) was used to classify the lodging grade of maize. The results provide a basis for the investigation of the crop lodging grade by UAV high-resolution images.

## 2. Materials and Methods

### 2.1. Overview of the Research Area

**Figure 1:**
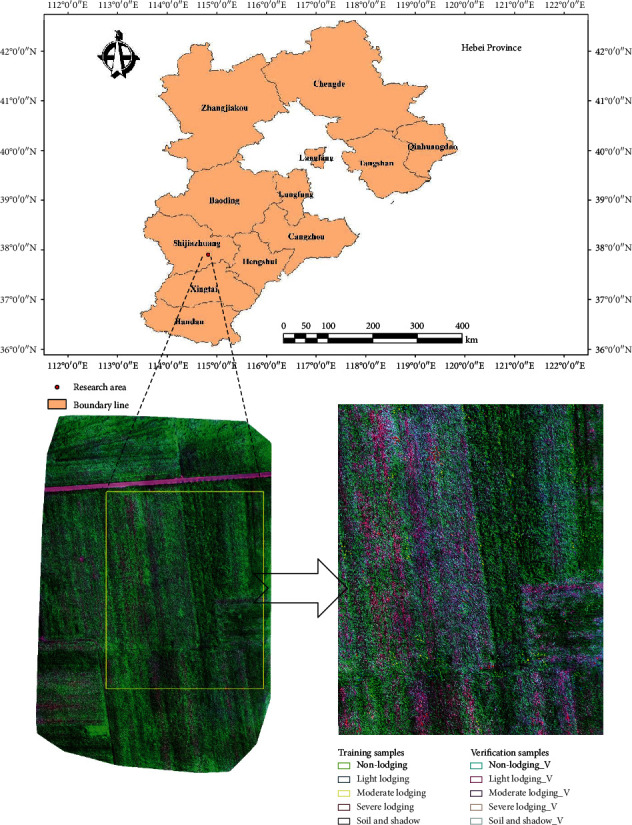
Geographical location and overview of the research area (ROI is the yellow frame) and distribution of training and verification samples.

The research area is located in Gaocheng, Shijiazhuang City, Hebei Province, China, with the geographical coordinates of 37°51′N–38°18′N, 114°39′E-114°59′E (Figure 1). Gaocheng is located in the North China Plain and has a warm temperate semihumid continental monsoon climate, characterized by cold winters and hot summers. The territory has four distinct seasons, with an average annual temperature of 12.5°C, an average annual rainfall of 494 mm, and an annual sunshine duration of 2711.4 hours. The cultivated land area of Gaocheng is about 549.02 km^2^. The crop planting is an annual double-cropping system. It is a wheat-maize rotation area. The maize variety in the research area is *Zhengdan 958* which is widely planted in the Huang-Huai-Hai area. The growth period of maize is about 96 days, the plant type is compact, the plant height is about 246 cm, the ear position is about 110 cm, and it is resistant to disease, lodging, and drought. Summer maize planting is generally completed around June 20th and harvested in October of the same year.

### 2.2. Data Acquisition

On September 5, 2018, there were severe winds in Gaocheng, and local wind speeds reached grade 6, which led to a large area of maize lodging during the maize filling period. The data in this study were derived from maize plots with different lodging grades acquired by the UAV platform at 2:00 pm on September 10, 2018.

A Parrot Sequoia camera served as a multispectral sensor for acquiring images mounted on a DJI Spreading Wings S1000. The single arm length of the UAV is 386 mm, the net weight of the UAV is about 4 kg, the weight of the carrier is 6 kg, and the duration of imaging is 15-20 min. The Parrot Sequoia camera consists of four spectral channels of green, red, red edge, and near-infrared (Table 1) with a global positioning system (GPS) and irradiance sensors. On the day of data acquisition, the weather was clear and calm. Radiometric calibration images obtained with the Parrot Sequoia camera were captured using a calibrated reflectance panel on the ground before and after each flight. The image resolution was 1280 × 960 pixels. The flight altitude was set at 60 m above the ground. The forward and lateral overlap was 80%. Flight speed was set to 6 m per second. There were eight routes, and a total of 271 multispectral images were collected per band.

### 2.3. Image Preprocessing

UAV multispectral images are preprocessed as follows: (1) Image screening: poor quality images during the UAV take-off and landing are deleted to decrease the number of images and ensure image quality. (2) Image stitching: 240 groups of screened images were stitched using the agricultural multispectral template in the Pix4D mapper software (version 4.0, Lausanne, Switzerland) [24, 25]. (3) Image clipping: due to the large flying area of the UAV, the size of the region of interest (ROI) was approximately 199.23m × 159.20m (Figure 1) and contained different lodging types.

The Sequoia camera combined with Pix4D software allows for better evaluation of collected data. The specific steps of mosaicking multispectral images using Pix4D software [26] are as follows: (1) Creating a new project. (2) Importing all discrete band imagery (green, red, red edge, and near-infrared imagery) from the flight. This includes the images of the calibration target. (3) Choosing the agricultural multispectral processing template. (4) Starting processing, including initial processing, point cloud and mesh, DSM (digital surface model), orthomosaic, and index. (5) Generating the orthomosaic imagery. Pix4D agricultural multispectral processing template automatically performs radiometric calibration [27, 28], which is to convert the digital number (DN) of ground object into absolute reflectance data.

The multispectral image geographic coordinate system was GCS_WGS_1984 and the projected coordinate system was UTM_Zone_50N and consisted of green, red, red edge, and near-infrared with a spatial resolution of 0.05 m.

### 2.4. Analysis Method

**Figure 2:**
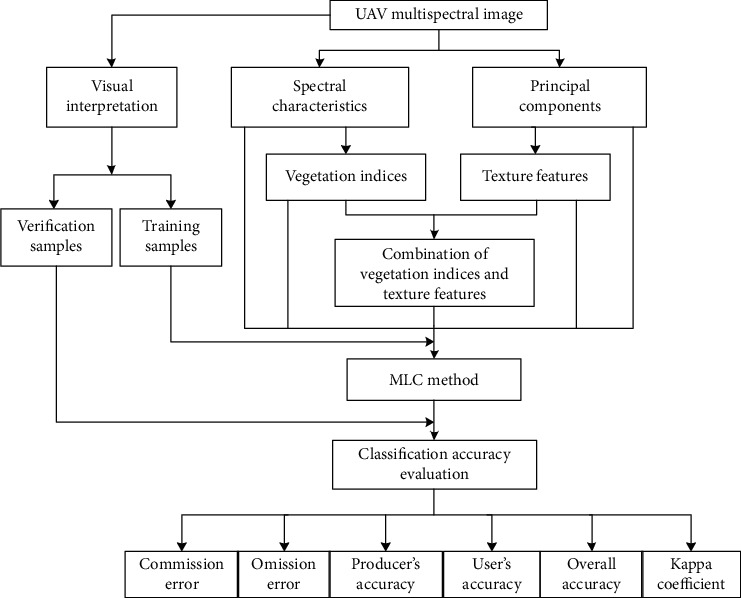
The workflow of maize lodging grade classification.

The spectral reflectance of maize with different lodging grades is various in four bands. We analyzed the spectral characteristics of maize with different lodging grades and constructed vegetation indices. At the same time, we extracted principal components of the UAV multispectral image and analyzed texture features. Five features are extracted; they are spectral characteristics, principal component, texture features, vegetation indices, and the combination of vegetation indices and texture features. The MLC [29, 30] supervised classification method was used to classify lodging grades combined with training samples based on the above five features. The classification accuracy was evaluated using a confusion matrix with verification samples. A method for extracting information regarding maize with different lodging grades based on UAV multispectral images is preferred. The workflow of maize lodging grade classification is shown in Figure 2.

### 2.5. Evaluation of Accuracy

Accuracy evaluation is an important and essential step in the classification process [31], which is to quantitatively determine how effectively pixels were grouped into the correct feature classes in the area. In order to verify the effectiveness of the classification results of different features, we need to use the verification samples to evaluate the classification results after the implementation of the supervised classification. The most common method used to evaluate the classification accuracy of remote sensing images is to establish a confusion matrix [32] for statistical analyses, which provides the classification accuracy for the population and each category. Furthermore, the accuracy of the classification results is displayed in a confusion matrix, which is a basic, intuitive, and simple method for measuring a classification model. The classification results were evaluated using the confusion matrix combined with the maize verification samples. Six indicators (Table 2) were used to evaluate the classification accuracy: producer's accuracy, user's accuracy, commission error, omission error, overall accuracy, and Kappa coefficient.

## 3. Monitoring Maize Lodging Grade

### 3.1. Definition of Maize Lodging Grades

**Figure 3:**
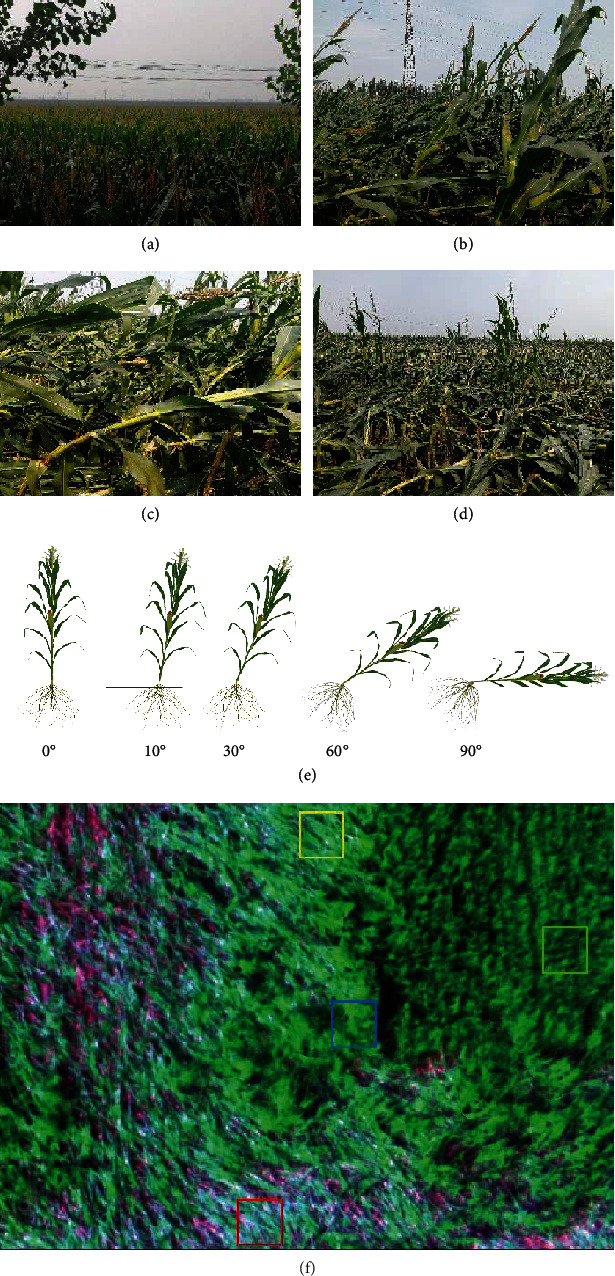
(a–d) are field survey photos of maize lodging: (a) is nonlodging, (b) is light lodging, (c) is moderate lodging, and (d) is severe lodging. (e) is a schematic diagram of maize with different lodging angles during the filling stage. (f) is an enlarged view of a part of the UAV multispectral image with different lodging grades. The green is nonlodging, the blue is light lodging, the yellow is moderate lodging, and the red is severe lodging.

Because the spatial resolution of the UAV multispectral image reached centimeters in our study, it is possible to distinguish maize with different lodging grades visually. Based on previous knowledge and field observation, nonlodging and three types of lodging were observed in the research area; lodging could be divided into light, moderate, and severe grades (Figure 3). Nonlodging indicates that the maize plants maintain an upright growth state; that is, the inclination angle (between the maize plant and the vertical line) of maize is 0°-10°. This is due to the strong self-resilience of maize, and the maize with slight lodging can recover quickly in a short time. Light lodging indicates that the inclination angle is 10°-30°; the plant is bent and has some ability for self-recovery. Moderate lodging refers to the plant with an inclination angle of 30°-60° and partially exposed maize stem. Severe lodging indicates that the inclination angle is 60°-90°; the plant falls so close to the ground, the stem is completely exposed, some stems are broken, and some of the lower leaves have dried up.

### 3.2. Training and Verification Samples

The smallest unit of a remote sensing image is a pixel. Image classification is defined as the process of categorizing all pixels in an image or raw satellite data to obtain a given set of labels [35]. Maximum likelihood supervised classification is a method in which the analyst defines representative small areas called training sites for each category on the image. The software then uses these training sites and applies them to the entire image. The delineation of training areas representative of a category is most effective when an image analyst has experience with the spectral properties of the category [35, 36].

The spatial resolution of the UAV multispectral image reaches a centimeter level which is 5 cm. Different lodging types can be identified from the image by visual interpretation. Visual interpretation refers to the process of extracting and analyzing the ground information contained in remote sensing images through human eye observation [37, 38]. Its advantage is that it can make full use of prior knowledge, and its interpretation accuracy is generally high. Visual interpretation was used to randomly select the maize sites with different lodging grades as the training and verification samples in the ROI based on prior knowledge, field investigation, and spectral reflectance. Because the samples are different in size and the basic classification unit is the pixel, we counted the pixel numbers of training and verification samples in ENVI 5.3 software (Table 3).

### 3.3. Spectral Reflectance Variation

**Figure 4:**
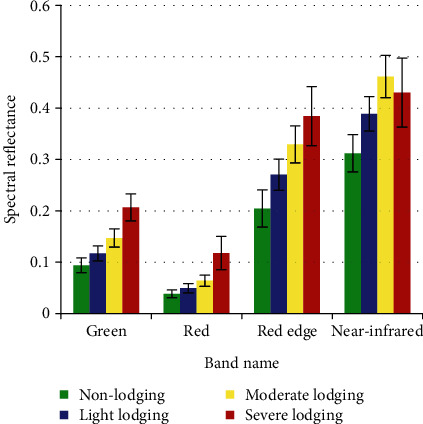
Spectral reflectance variation of maize with different lodging grades.

The agricultural multispectral Parrot Sequoia camera has four spectral channels, including green, red, red edge, and near-infrared. The means and standard deviations of maize reflectance with different lodging grades of training samples were extracted and calculated (Figure 4). Because the multispectral images have been corrected by radiometric calibration in Pix4D software, the actual reflectance data of the surface object have been obtained. The four spectral reflectances are calculated by the average of all pixels of training samples with different lodging types; we extracted it directly from the statistical function of ENVI 5.3 software.

The spectral reflectance of the same lodging type is consistent with the vegetation spectral curve. The spectral reflectance with different lodging grades varied consistently in four bands, which were lower in red and higher in near-infrared. In different bands, the spectral reflectance of lodging was higher than that of nonlodging, and the more serious the lodging, the higher the spectral reflectance [39] (Figure 4). The main reason is that lodging changes the structure and shape of the crop population and causes the change of canopy spectral reflectance. The more serious the lodging, the more stem exposure, and the reflectance of the stem is higher than that of leaf [39]. Severe lodging caused the maize stems to break and the lower leaves to dry; thus, the reflectance is slightly lower than moderate lodging in near-infrared. After lodging, the original canopy structure collapsed, the proportion of stems increased significantly, and the proportion of leaves decreased, which caused the reflectance of different lodging grades to increase [40, 41]. In four bands, spectral reflectance of lodging maize varies most in the near-infrared band compared to nonlodging maize [42] (Figure 4, Table 4).

**Figure 5:**
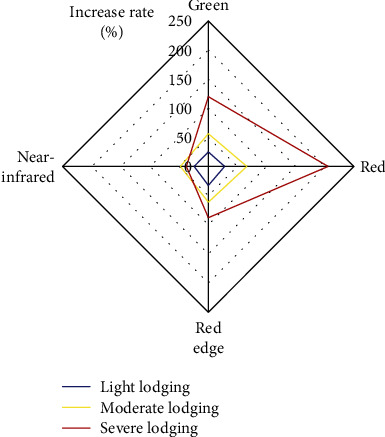
Increase rate in the spectral reflectance of maize with different lodging grades.

Compared with nonlodging maize, the spectral reflectance of light lodging increased by 24.54–32.28%, that of moderate lodging increased by 47.84–66.03%, and that of severe lodging increased by 37.83–205.79%. This showed that the more serious the lodging, the higher the increase of spectral reflectance. Under the same lodging grade, the increase rate in the visible band was larger than that in the near-infrared band [39] (Figure 5).

### 3.4. Construction of Vegetation Indices

The vegetation index is a linear or nonlinear combination of two or more characteristic bands, which reflects the relative abundance and activity of green vegetation. The main purpose of establishing vegetation indices is to effectively synthesize relevant spectral signals, enhance vegetation information, and reduce non-vegetation-related information. The vegetation index mainly reflects the difference between the visible and near-infrared bands and the soil background. Each vegetation index can be used to quantitatively describe the growth status of vegetation under certain conditions.

The normalized differential vegetation index (NDVI) [43] is currently the most widely used index. Due to the easy saturation of the red band and neglect of the green band, Gitelson et al. [44] proposed the green normalized vegetation index (GNDVI). Studies have shown that the green band is closely related to vegetation parameters [45, 46]. The soil-adjusted vegetation index (SAVI) modified the sensitivity of NDVI to soil background and reduced its impact. The renormalized difference vegetation index (RDVI) provides the advantages of both NDVI and difference vegetation index (DVI), which can be used for vegetation with different coverage. The calculation formulas of the four vegetation indices are displayed in Table 5.

**Figure 6:**
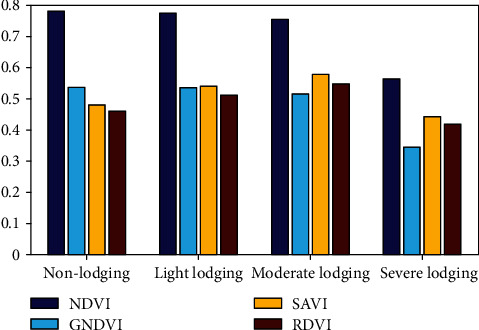
Variance of vegetation indices.

The variance of vegetation indices with different lodging grades is shown in Figure 6. The change trend of different vegetation indices is various under lodging stress. The more serious the lodging, the smaller the NDVI, which is consistent with the results of previous studies [39]. GNDVI decreases with the increase of lodging grades, because the relative increase rate of spectral reflectance in the visible band was larger than that of the near-infrared band after lodging. Compared with nonlodging, the NDVI and GNDVI reductions of light lodging are smaller, because we collected UAV data on the 5th day after lodging, and the light lodging maize returned to normal growth.

SAVI and RDVI decreased in severe lodging which were lower than those of nonlodging after increasing slightly with the increase of lodging grades. SAVI values range from -1 to 1. The lower the green vegetation coverage, the lower the SAVI value [49]. The nonlodging maize plants are erect, and the canopy spectra are mainly from the upper leaves. And the leaves shaded each other, which have an influence on the canopy spectra. Compared with nonlodging plants, the vegetation coverage of light and moderate lodging increased, and the vegetation coverage of moderate lodging was higher than that of light lodging. Therefore, the order of SAVI value is moderate lodging > light lodging > nonlodging. For severe lodging, due to the lower leaves of the plants turn yellow and dry, the soil background was exposed and the vegetation coverage decreased. The vegetation coverage of severe lodging is lower than that of the nonlodging, so the SAVI value is lower than the nonlodging maize.

NDVI decreased with the increase of lodging severity, and RDVI decreased after increasing, indicating that RDVI is mainly affected by DVI, and DVI is extremely sensitive to changes in soil background [50]. The spectral reflectance of vegetation in near-infrared is higher than that in the red band. In nonlodging, light, and moderate lodging, the difference between near-infrared and red band increases with the increase of lodging grades. In the case of severe lodging, the reflectance in near-infrared decreases due to the influence of soil, so RDVI decreases and is smaller than that of nonlodging.

### 3.5. Principal Components and Texture Features

PCA can remove redundant information between bands while compressing multiband information into several, more efficient bands [51, 52]. There are four bands in the multispectral image; in order to avoid data redundancy, PCA was performed to obtain the first few principal components which contained more information. The first two principal components contain 96.86% information of all bands and could be used to extract texture features in this study.

The texture feature involves the extraction and analysis of spatial distribution patterns of gray grade in an image, which is widely used in image classification and target recognition [53–55]. Texture feature parameters are extracted to obtain qualitative or quantitative descriptions of texture by certain image processing technology. The gray level cooccurrence matrix (GLCM) is a comprehensive analysis of its local features and arrangement based on pixels. Haralick et al. [56] extracted and analyzed image texture features using GLCM and derived 14 parameters for quantifying texture features. The GLCM reflects the distribution characteristics of the gray level and presents changes in the repetition, alternation, or specific rules in the spatial range, which is a second-order statistical feature of the grayscale change of the image. The extraction of 8 texture features (Table 6) based on GLCM uses the first two principal components. The 8 texture features were mean, variance, homogeneity, dissimilarity, contrast, entropy, second moment, and correlation. After comparative analysis, the filtering window was set to 7 × 7, the spatial correlation matrix offset *X* and *Y* were 1, and the grayscale quality was 64.

**Figure 7:**
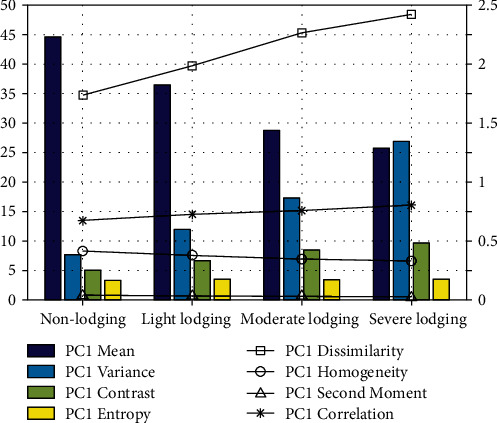
Variation of texture features with different lodging grades.

The texture feature parameters of the first principal component were used as an example to analyze the variation characteristics of different lodging grades (Figure 7). Since the parameters differ in magnitude, the mean, variance, contrast, and entropy were represented by a bar chart, and the homogeneity, dissimilarity, second moment, and correlation were represented by a line chart. It was clear that the various parameters differed when the lodging grades were different. The mean, homogeneity, and second moment decreased with increasing lodging grades, while variance, dissimilarity, contrast, entropy, and correlation increased with increasing lodging grades. Texture features reflect the spatial variability of spectral changes in every band. The texture features of different bands are usually various, so texture features have different abilities to distinguish different lodging grades.

## 4. Results

### 4.1. Classification Results

**Figure 8:**
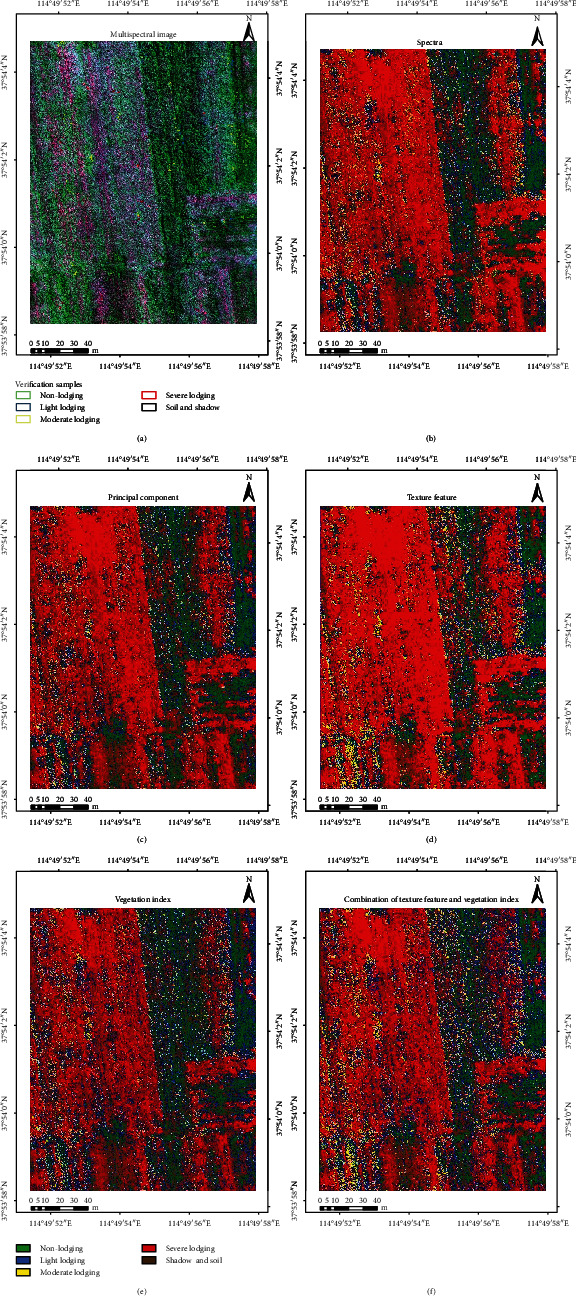
(a) The UAV multispectral image: the band combination was red, near-infrared, and green. (b) Multispectral image classification. (c) Classification based on the first two principal component features. (d) The texture feature classification. (e) Results of the vegetation index classification. (f) Combination of texture features and vegetation index classification.

Based on the spectral features, principal component features, texture features, vegetation indices, and the feature combination, the MLC method was used to classify lodging grades (Figure 8). Figure 8(a) presents the original UAV multispectral image, which is displayed as a false color combination of red, near-infrared, and green bands. The spatial resolution of the image was up to 0.05 m. Based on prior knowledge and agronomic experience, the different lodging grades, the shadow of maize plants, and the soil exposed by lodging can be clearly distinguished from the image, and soil and shadow were combined into one category. It was clear that the area of maize lodging was larger due to the influence of strong wind, and the area of severe lodging was the largest, being mainly distributed in the left half of the ROI (Figure 8(a)). It was also clear that the severe lodging area is the largest, and all areas are located in the left-central area of the ROI (Figures 8(b)–8(f)). The ROI area was about 31718.39 m^2^; we counted the area of each lodging type (Table 7). The results were slightly different but showed that the area of severe lodging was the largest and moderate lodging was the smallest.

**Figure 9:**
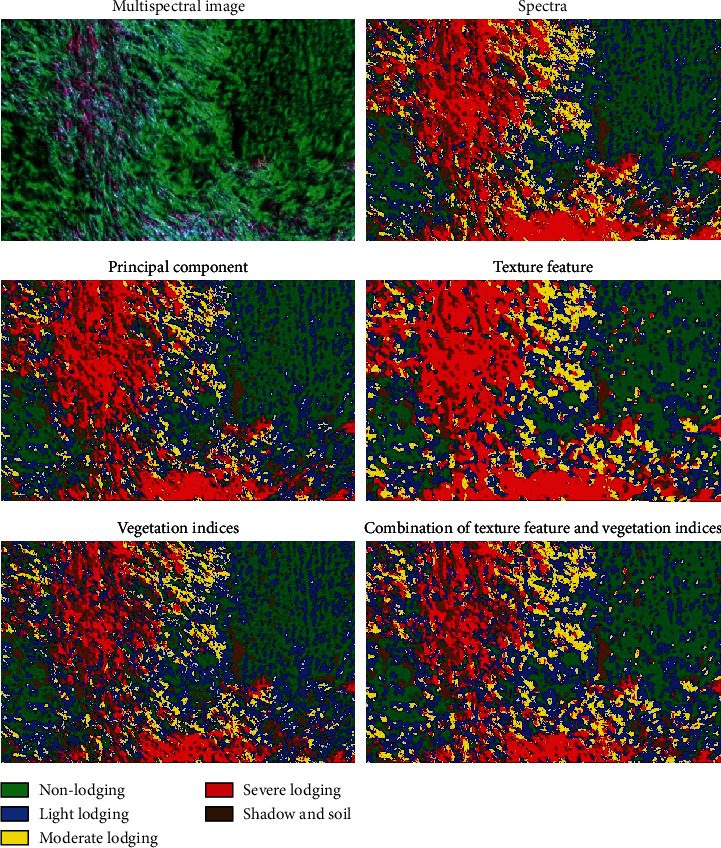
Partial enlargement of classification results which contain ground truth with different lodging types.

The ground truth was used to verify the classification accuracy; we extract the partial enlargement of classification results which contain ground truth with different lodging types (Figure 9). For nonlodging, the result of texture features and combination of texture features and vegetation indices is right; in other classification results, there is error phenomenon of classifying nonlodging into light lodging. For light lodging, all the classification results showed that some of the pixels were classified into nonlodging and moderate lodging. For moderate lodging, all the classification results showed that some of the pixels were classified into nonlodging, light lodging, and severe lodging. For severe lodging, there is also a phenomenon of misclassification. Some pixels were classified into light lodging, moderate lodging, and shadow and soil.

### 4.2. Validation

The classification of the maize lodging grade was verified using the confusion matrix combined with verification samples. The classification accuracy of 6 evaluation indicators for each category is shown in Table 8. From the perspective of producer's and user's accuracy, the accuracy of the shadow and soil class was the highest, followed by severe lodging, and light lodging was lowest. Because the image characteristics of shadow and soil are far from those of normal and lodging maize in ROI, they are easy to identify. Severe lodging caused most or all stems of maize to be exposed as a result of falling to the ground. In addition, the reflectance of maize leaves and stems as well as the image characteristics is differed, making them easy to recognize. From the perspective of commission error, light lodging and moderate lodging are most serious, which may be due to the small difference among the light, moderate lodging, and nonlodging types. The omission error is large in the case of light lodging, which is divided into other lodging types. This is because of some overlap of characteristic information among the different lodging types.

The overall accuracy of spectrum features, principal component features, vegetation indices, texture features, and the feature combination was 83.58, 84.54, 83.40, 85.05, and 86.61%, respectively, and the Kappa coefficients were 0.7947, 0.8068, 0.7925, 0.8131, and 0. 8327, respectively. The overall accuracy of the spectrum features reached 83.58%, indicating that visual interpretation is reliable to identify the different lodging grades with high-resolution images. The overall accuracy of the first two principal components is about 1% higher than that of the spectral image. PCA focuses on the various information features of different lodging grades in fewer bands, thus avoiding data redundancy. Vegetation indices were 0.18% lower than the spectral feature, which may be due to the vegetation indices being less, along with the small differentiation between various lodging grades of maize (especially between light and moderate lodging). The overall accuracy of texture features was higher than those of spectral and principal component features, mainly because the spatial resolution of UAV image is high and the texture features reflect the homogeneous phenomenon in the image. Additionally, the phenomena of “same object different spectra” and “different objects same spectra” in spectral feature recognition can be avoided, and classification accuracy is improved. The overall accuracy and Kappa coefficient of the feature combination were the highest, which are higher than those of single feature. The results show that the method based on feature combination can significantly improve the classification accuracy of maize lodging grades during the filling stage.

## 5. Discussion

Monitoring crop lodging needs to simultaneously determine the grades and area in order to better evaluate the yield and economic loss caused by lodging stress [60, 61]. After lodging, the canopy structure of maize changed greatly, mainly regarding the ratio of stem to leaf in the detection field of view and the light exposure conditions of each part of the plant; thus, there is significant difference in spectral reflectance of different lodging types. The more serious the lodging stress, the more exposed the stem, and the higher the spectral reflectance. We proposed a method based on the UAV multispectral image to classify the lodging grades of maize during the filling stage.

Timely acquisition of crop lodging grades and area is of great significance for the assessment of yield loss. The development of remote sensing provides an important method to monitor crop lodging [6, 62]. However, satellite data can be affected by clouds, the revisiting time is long, and the spatial resolution is low. There is a mixed pixel phenomenon, and the cost of remote sensing data with high spatial resolution is also high, which leads to large uncertainty in crop lodging monitoring. Presently, there is a focus on research on crop lodging monitoring based on the UAV platform with digital and multispectral cameras. The UAV imaging system is fast, nondestructive, low-cost, and unaffected by the atmosphere [10–16, 63]. The data in the present study were derived from UAV multispectral images, which contain four bands: green, red, red edge, and near-infrared. Compared with an RGB image [12–15], the spectral characteristics of maize in different bands can be used to qualitatively distinguish different lodging grades. At the same time, the vegetation indices can be constructed using multiple bands. The vegetation index reflects the activity and physiological characteristics of green vegetation (for example, chlorophyll content and photosynthesis intensity), which can be used to quantitatively explain the growth status of crops [64, 65]. The variation characteristics of vegetation indices differ with different lodging grades. The texture features can also be extracted based on the GLCM, which reflects the spatial variability of spectral changes of ground objects [66]. The various texture parameters of maize with different lodging grades differ. In addition, many studies have been performed to identify crop lodging and extract crop lodging areas by UAV; however, they only classified crop to lodging and nonlodging or predicted the occurrence probability of lodging [12, 14, 15]. In fact, the influence of different lodging grades on crops is usually various. After crops are subjected to lodging stress, photosynthesis in leaves is suppressed to varying extents, and physiological and biochemical parameters are also changed [67–69]. Thus, crop yield is affected; when the lodging is light, it will lead to the reduction of crop yield, and when the lodging is serious, it will lead to the failure of crop harvest; therefore, a more detailed division of crop lodging grades is needed.

In this study, the MLC method was used to classify the lodging grades of maize during the filling stage. Due to the lack of field survey data and the high spatial resolution of 0.05 m, combined with prior agronomic knowledge, it was possible to visually distinguish different lodging grades. The overall accuracy of the multispectral image was 83.58%, and the Kappa coefficient was 0.7947, indicating that the training samples for visual interpretation are reliable for identifying the lodging grades of maize. The overall accuracy of the combination of texture features and vegetation indices was 3.03% higher than that of the spectral feature, indicating that the feature combination is better for classification of lodging grades.

RGB images have been used for a long time to observe crops in order to compile a history of the fields and to analyze reparceling. The RGB camera only obtains the information of red, green, and blue bands, and all of them are in the visible band. Compared with RGB data, multispectral data contains not only the visible band but also the near-infrared band. When the vegetation is stressed, the chlorophyll content and water content will change, although this change appears synchronously in visible and near-infrared band, but the change is more obvious in the near-infrared [20]. Our previous studies [40–42] have shown that crop lodging causes the collapse of the original canopy structure and disrupts the distribution of leaves; some or a considerable number of leaves are pressed or covered. The more serious the lodging degree, the stem-leaf ratio of canopy also increases. The higher the exposure ratio of the canopy stem is, the higher the spectral reflectance is, and the change in near-infrared band is more significant than that in the visible band. Therefore, the near-infrared band provides important information in monitoring crop lodging.

Regarding the flight altitude, we refer to References [14, 28, 70, 71]. When the flying height is too high, the spatial resolution of the image will be low and the spectral information will be lost. When the flying height is too low and the spatial resolution is high, a maize plant will be divided into more categories; at the same time, the amount of image data will increase, resulting in data redundancy. In addition, the flight area will also become smaller. The high spatial resolution of 0.05 m ensures that there are all pure pixels, which avoids the interference caused by mixed pixels and omits the mixed pixel decomposition in image classification [72, 73]. Multispectral image classification is strongly affected by spatial resolution while the accuracy is not necessarily improved by the increase of the spatial resolution [74]. There exists an optimal resolution for each geographical entity, corresponding to its intrinsic spatial and spectral characteristics. Because the resolution of the UAV multispectral image was high and a pixel was used as the basic classification unit, the reflectance and characteristics of maize leaves and stems differed. Thus, the same maize is inevitably divided into two or more categories. Although the degree of stem exposure in maize was different, the spectra and other characteristics of different lodging grades were similar, which can easily lead to misclassification.

In the maize planting plot, we conducted a field survey of maize varieties and lodging types. The UAV flight plots we selected included different types of lodging, and the maize varieties were *Zhengdan 958*, which avoids the difference caused by maize varieties and the defect of the single lodging type in the same plot. The maize plant has strong growth ability and a certain recovery ability after lodging, which is various with different lodging grades [40, 41]. In different times of the same lodging type, coupled with the self-recovery of maize, the canopy structure will change greatly and the spectral reflectance will also change. In addition, the mechanism of plant recovery after lodging is a complex process, which is affected by many factors. The method proposed in this paper is effective to classify the lodging types of the data collected on the same day and avoids the influence of the reflectance of the same lodging type in different periods. In the next work, we will study the recovery of maize after lodging from multitime series data. Also, we will add field survey data, make comprehensive use of multisource remote sensing data, introduce more image features, combine different features, and use an object-oriented method and other classification methods to improve accuracy and identify the optimal method to classify maize lodging grades. Providing the relevant agricultural production departments and agricultural insurance companies with data on accurate losses caused by different grades of crop lodging will permit reasonable postdisaster remedial work and claim-settlement programs.

## 6. Conclusions

First, the spectral reflectance of maize with different lodging grades was extracted from UAV images using training samples, and the spectral characteristics of maize with different lodging grades were analyzed. Compared with nonlodging, the more serious of lodging grades, the higher the spectral reflectance, the greater the increase in spectral reflectance. Then, the principal components were extracted, the texture features based on GLCM were transformed, the four vegetation indices were constructed, and the texture features and vegetation indices were combined. The maize lodging grades of different features were classified by the MLC method. The results showed that the severe lodging area was the largest and was distributed in the left half of the field plot. Finally, based on the verification samples with different lodging grades, classification accuracy was evaluated using a confusion matrix. The overall accuracy of the spectral features was 83.58%, and the Kappa coefficient was 0.7947. The overall accuracy of the combination of texture features and vegetation indices was 86.61%, and the Kappa coefficient was 0.8327, and these values were 3.03% and 0.038 higher than those of the spectrum features, respectively. There is an advantage of quickly and accurately extracting the maize lodging grades based on feature combination of high-resolution UAV multispectral image.

## Figures and Tables

**Table 1:** Parrot Sequoia camera parameters.

Band name	Center wavelength (nm)	Bandwidth (nm)
Green	550	40
Red	660	40
Red edge	735	10
Near-infrared	790	40

**Table 2:** Evaluation index of classification accuracy.

Evaluation index	Formula	Description	References
Producer's accuracy	$\text{PA}\left(C_{i}\right)=\frac{\alpha_{ii}}{\sum_{i=1}^{N}\alpha_{ki}}\times 100\%$	Any pixel selected from the ground truth, the probability of which is the same as the classification result, is calculated by using the columns of the confusion matrix.	[32–34]

User's accuracy	$\text{UA}\left(C_{i}\right)=\frac{a_{ii}}{\sum_{i=1}^{N}a_{ik}}\times 100\%$	Any pixel selected from the classification result, and the probability that it is consistent with the ground truth, is calculated by using the rows of the confusion matrix.	[32–34]

Commission error	CE(*C*_*i*_) = 1 − UA(*C*_*i*_)	The pixels that belong to the ground truth but are not classified into the corresponding categories and are displayed in the columns of the confusion matrix.	[31, 34]

Omission error	OE(*C*_*i*_) = 1 − PA(*C*_*i*_)	The pixels that are divided into one category and actually belong to another, displayed in the rows of the confusion matrix.	[31, 34]

Overall accuracy	$\text{OA}\left(C_{i}\right)=\frac{1}{n}\overset{N}{\underset{i=1}{\sum }}\alpha_{kk}\times 100\%$	For each random pixel, the probability that the classification result is consistent with the actual type of the corresponding area on the ground truth.	[32–34]

Kappa coefficient	$\text{K}\left(C_{i}\right)=\frac{n\sum_{i=1}^{N}\alpha_{ii}-\sum_{i=1}^{N}\left(\alpha_{ik}\times \alpha_{ki}\right)}{n^{2}-\sum_{i=1}^{N}\left(\alpha_{ik}\times \alpha_{ki}\right)}$	Kappa analysis is an indicator to determine the coincidence or accuracy between two images. Kappa coefficient is -1-1, but usually between 0 and 1. The closer to 1, the higher the precision.	[32–34]

**Table 3:** The pixel numbers of training and verification sample.

Lodging grade	Training sample	Verification sample
Nonlodging	7484	6472
Light lodging	7461	6448
Moderate lodging	7461	6448
Severe lodging	7495	6434
Soil and shadow	7485	6423

**Table 4:** Variation value in spectral reflectance.

Lodging grade\band name	Green	Red	Red edge	Near-infrared
Nonlodging	0	0	0	0
Light lodging	0.0234	0.0108	0.0660	0.0766
Moderate lodging	0.0531	0.0255	0.1249	0.1493
Severe lodging	0.1126	0.0794	0.1798	0.1181

**Table 5:** Calculating formula for various vegetation indices.

Vegetation index	Calculating formula	Reference
NDVI	$\text{NDVI}=\frac{\rho_{\text{NIR}}-\rho_{\text{RED}}}{\rho_{\text{NIR}}+\rho_{\text{RED}}}$	[43]

GNDVI	$\text{GNDVI}=\frac{\rho_{\text{NIR}}-\rho_{\text{GREEN}}}{\rho_{\text{NIR}}+\rho_{\text{GREEN}}}$	[44]

SAVI	$\text{SAVI}=\frac{\left(1+L\right)*\left(\rho_{\text{NIR}}-\rho_{\text{RED}}\right)}{\left(\rho_{\text{NIR}}+\rho_{\text{RED}}\right)+L}$	[47]

RDVI	$\text{RDVI}=\sqrt{\text{NDVI}*\text{DVI}}$	[48]

Note: *L* = 0.5 in the area of medium vegetation cover.

**Table 6:** Parameters of texture feature.

Parameters	Formula	References
Mean	$\mu_{i}=\overset{N-1}{\underset{i,j=0}{\sum }}i\left(P_{i,j}\right)$ $\mu_{j}=\overset{N-1}{\underset{i,j=0}{\sum }}j\left(P_{i,j}\right)$	[57–59]
Variance	${\sigma_{i}}^{2}=\overset{N-1}{\underset{i,j=0}{\sum }}P_{i,j}{\left(i-\mu_{i}\right)}^{2}$ ${\sigma_{j}}^{2}=\overset{N-1}{\underset{i,j=0}{\sum }}P_{i,j}{\left(j-\mu_{j}\right)}^{2}$	
Homogeneity	$\overset{N-1}{\underset{i,j=0}{\sum }}\frac{P_{i,j}}{1+{\left(i-j\right)}^{2}}$	
Dissimilarity	$\overset{N-1}{\underset{i,j=0}{\sum }}P_{i,j}\left|i-j\right|$	
Contrast	$\overset{N-1}{\underset{i,j=0}{\sum }}P_{i,j}{\left(i-j\right)}^{2}$	
Entropy	$\overset{N-1}{\underset{i,j=0}{\sum }}P_{i,j}\left(-\log P_{i,j}\right)$	
Second moment	$\overset{N-1}{\underset{i,j=0}{\sum }}{P_{i,j}}^{2}$	
Correlation	$\overset{N-1}{\underset{i,j=0}{\sum }}P_{i,j}\left[\frac{\left(i-\mu_{i}\right)\left(j-\mu_{j}\right)}{\sqrt{\left({\sigma_{i}}^{2}\right)\left({\sigma_{j}}^{2}\right)}}\right]$	

**Table 7:** The area of different lodging grades (m^2^).

Lodging grade	Spectra	Principal component	Texture features	Vegetation indices	Combination of texture features and vegetation indices
Nonlodging	4689.19	5628.52	4228.78	5448.82	4503.47
Light lodging	4498.63	4923.99	4229.87	6728.50	6672.33
Moderate lodging	2648.37	1849.09	2459.68	2153.50	2888.99
Severe lodging	12521.23	12341.78	16513.41	9385.36	10780.50
Shadow and soil	7360.97	6975.01	4286.65	8002.21	6873.10

**Table 8:** Evaluation of classification accuracy (%).

Evaluation index	Lodging grade	Spectra	Principal component	Vegetation index	Texture feature	Combination of texture feature and vegetation index
Producer's accuracy	Nonlodging	78.35	78.11	74.57	88.49	86.40
	Light lodging	74.12	75.43	77.25	69.07	75.11
	Moderate lodging	80.20	82.92	78.68	85.64	84.85
	Severe lodging	86.57	87.50	87.47	88.73	88.56
	Shadow and soil	98.79	98.88	99.19	93.43	98.27

User's accuracy	Nonlodging	84.17	85.00	85.87	86.17	89.90
	Light lodging	71.72	71.66	69.58	80.56	80.96
	Moderate lodging	77.75	79.19	79.12	76.10	77.39
	Severe lodging	86.48	89.29	86.11	84.92	86.99
	Shadow and soil	98.48	98.74	98.11	98.73	98.52

Commission error	Nonlodging	15.83	15.00	14.13	13.83	10.10
	Light lodging	28.28	28.34	30.42	19.44	19.04
	Moderate lodging	22.25	20.81	20.88	23.90	22.61
	Severe lodging	13.52	10.71	13.89	15.08	13.01
	Shadow and soil	1.52	1.26	1.89	1.27	1.48

Omission error	Nonlodging	21.65	21.89	25.43	11.51	13.60
	Light lodging	25.88	24.57	22.75	30.93	24.89
	Moderate lodging	19.80	17.08	21.32	14.36	15.15
	Severe lodging	13.43	12.50	12.53	11.27	11.44
	Shadow and soil	1.21	1.12	0.81	6.57	1.73
Overall accuracy		83.58	84.54	83.40	85.05	86.61
Kappa coefficient		79.47	80.68	79.25	81.31	83.27
